# Weight and weight control behaviors during long‐term endometrial cancer survivorship: Results of the Laparoscopic Approach to Cancer of the Endometrium long‐term follow‐up study

**DOI:** 10.1002/cam4.4032

**Published:** 2021-06-18

**Authors:** Monika Janda, Peta Forder, Val Gebski, Saira Sandjia, Nigel Armfield, Andreas Obemair

**Affiliations:** ^1^ Faculty of Medicine Centre for Health Services Research The University of Queensland Brisbane Queensland Australia; ^2^ HMRI Public Health Program School of Medicine and Public Health The University of Newcastle Newcastle Australia; ^3^ NHMRC Clinical Trials Centre The University of Sydney Sydney New South Wales Australia; ^4^ Queensland Centre for Gynaecological Cancer Brisbane Queensland Australia; ^5^ Faculty of Medicine Centre for Clinical Research The University of Queensland Brisbane Queensland Australia

**Keywords:** discontent with weight, endometrial cancer, long‐term follow‐up, survivorship, weight control

## Abstract

**Background:**

Overweight or obesity is common in endometrial cancer (EC). This study aimed to examine sociodemographic, clinical, and psychosocial characteristics associated with being discontent with current weight and use of weight control methods among long‐term EC survivors.

**Methods:**

Women diagnosed with early‐stage EC who participated in the Laparoscopic Approach to Cancer of the Endometrium (LACE) trial (*n* = 516) were invited to complete a long‐term follow‐up survey at least 4.5 years after treatment. Chi‐square test and multivariate logistic regression models adjusted for time since surgery were used to determine factors associated with being discontent with current weight.

**Results:**

On average 9 years after surgery, 190/259 (73%) of participants were currently discontent with their weight, and 146 (56%) had used one or more weight loss methods during the past 12 months. Women who were discontent with their weight were more likely to be younger than 70 years (*p *< 0.000), and used one or more weight loss methods ever or during the past 12 months (*p* < 0.000). Among the weight loss methods used, exercise (40.1%), meal reductions (52.7%), or fat/sugar reductions (48.5%) were much more commonly reported than fasting (2.6%) or designated weight loss programs (2.3%).

**Conclusions:**

Our study provides evidence that the majority of long‐term EC survivors in this clinical trial population are discontent with their weight and over half continue to use multiple methods to lose weight each year. These data indicate that health professionals and lifestyle educators need to assess weight issues, and develop a tailored plan to address the specific needs of long‐term survivors to assist them become content with their weight after treatment for EC.

## Graphical abstract

On average nine years after their initial endometrial cancer (EC) treatment, three quarters of women reported they were not content with their body weight, and more than half had used one or more weight loss programs or methods over the past 12 months. Younger age and greater usage of weight loss methods were associated with being discontent with body weight. Exercise and reducing meals, fats or sugar were common behavioral strategies, while fewer women used fasting, commercial programs, slimming products or medications to lose weight.

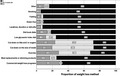

## 1 INTRODUCTION

Endometrial cancer (EC) is the most common subtype of uterine cancer, which is the fifth most common cancer in women in developed countries, with an estimated worldwide incidence of 382,069 new cases per year.^1^, ^2^ The highest age‐standardized incidence rates in 2018 were estimated at 19.1 and 22.2 per 100,000 in North America and Europe, respectively.^2^, ^3^ Evidence suggests that this is attributable to the high overall prevalence of obesity and metabolic syndromes in these regions,^4^ which are the established risk factors for EC.^5^

Most patients present with early stage disease due to the appearance of symptoms, especially irregular or post‐menopausal bleeding, early in the course of EC development.^6^ Despite substantial prognostic differences between histological subtypes, the overall 5‐year survival of EC is approximately 80%^7^ when standard treatment of total hysterectomy and bilateral salpingo‐oophorectomy is received.^8^ Surgery is most commonly done by total laparoscopic hysterectomy (TLH), which has been shown to be associated with equivalent survival, fewer postoperative complications and better quality of life (QoL) compared to total abdominal hysterectomy (TAH).^9^, ^10^, ^11^ Although survival outcomes are good, women presenting with early stage EC are typically overweight or obese, which may independently impact women's longer‐term physical and psychosocial well‐being.^12^ Epidemiologic studies have shown that EC survivors who are obese have poorer QoL and are at increased risk of morbidity and mortality compared to their non‐obese counterparts.^13^, ^14^, ^15^, ^16^ There is evidence that many women continue to gain weight after surgical treatment, and postoperative weight gain may have a negative effect on survival.^17^ Despite this major impact of excessive weight on health and QoL outcomes, data regarding the disease‐specific and overall health benefits of weight loss programs following EC treatment are sparse, and patients’ own efforts at losing weight are rare.^18^, ^19^ As part of improving patient management and informing programs to optimize ongoing health after EC treatment, identifying factors that contribute to women's perception of their own weight and weight loss options may enhance supportive care efforts in the future. Using data from the long‐term follow‐up of the Laparoscopic Approach to Cancer of the Endometrium (LACE) trial, it was the objective of this study to explore the association between self‐reported discontent with body weight, sociodemographic, clinical, and psychosocial characteristics and weight control behaviors among women who underwent treatment for apparent Stage I EC more than 4.5 years ago.

## 2 METHODS

### 2.1 Study population

The Laparoscopic Approach to Cancer of the Endometrium (LACE) trial was a phase III international multicenter randomized clinical trial designed to test the hypothesis that TLH is associated with equivalent disease‐free survival compared to the standard treatment of TAH among women with apparent Stage I EC. A total of 760 women with stage I EC from 20 tertiary gynecological cancer centers in Australia, New Zealand, and Hong Kong were randomized to either TAH or TLH between 7 October 2005 and 30 June 2010. The study design and several outcomes have been previously reported.^9^, ^10^, ^11^, ^20^, ^21^ After excluding women known to be deceased, withdrawn from the trial, lost to follow‐up (address unknown), or known to have a serious illness or recurrence, 516 of the initial 760 participants were invited to complete the long‐term, self‐reported follow‐up survey when the last patient enrolled had reached at least 4.5 years after surgery in 2016–2017.^22^

### 2.2 Sociodemographic and clinical characteristics

Patients’ age, employment status (retired or employed), and medical history were extracted from follow‐up data. Educational attainment (≤12 years vs. >12 years), marital status (living with or without partner), private health insurance (yes, no), country of birth (Australia, other), and hysterectomy type (TAH vs. TLH) were extracted from baseline and surgical assessment date.

### 2.3 Participants’ weight and weight lost program

This analysis used data stemming from questions assessing patients’ weight [How much do you weigh on the scales without clothes or shoes? answered in kg] and if patients were satisfied with their current body weight [“How much would you like to weigh?”]. Responses to the weight satisfaction question were classified as either content (“Happy as I am”) or discontent (“1–5 kg more,” “Over 5 kg more,” “1–5 kg less,” “6–10 kg less,” “11–15 kg less,” “16–20 kg less,” and “Over 21 kg less”). Patients were also asked to report on their use [Have you used any of these methods to lose weight or control your weight or shape?] of the following types of weight control methods: commercial programs, meal replacements or slimming products, exercise, reduced meal intake, reduced fats/sugars, low glycemic index (GI) diet, diet books, medications (laxatives, diuretics or diet pills), gluten‐free diet, fasting, smoking, or other methods. Responses for each method were recorded as “Never,” “Ever,” or “In the last 12 months.”

### 2.4 Other measurements

Participants also completed questionnaires to assess the following outcomes–

Hospital Anxiety and Depression scale (HADS): This is a 14‐item questionnaire used to determine anxiety and depression (7 items for each).^23^ Items are scored from 0 to 3 and eight items are reverse‐coded to form sub‐scales ranging from 0 to 21.^24^, ^25^ Each sub‐scale is categorized by recommended cut‐off values into normal (0–7), borderline anxiety/depression (8–10), and anxiety/depression (11–21).^23^

Functional Assessment of Cancer Therapy–General population (FACT–GP): This validated quality of life measure consists of four subscales: physical well‐being (6 items), social well‐being (5 items), emotional well‐being (4 items), and functional well‐being (6 items).^26^ Each item for social and functional well‐being was rated from 0 (as “not at all”) to 4 (as “very much”), other items were reversed coded. Missing items for each measurement scales were replaced by computing the mean of answered items, if more than 60% of the items in a subscale had been answered. Higher scores indicated better quality of life.^27^

The Active Australia Survey: This validated scale measured physical activity by calculating total time (minutes) spent in walking, moderate, and vigorous (time x 2) physical activity in the last week.^28^ Women without any activity were considered as “sedentary.” Spending time in physical activity for at least 150 minutes/week and more than 150 minutes/week was classified as “insufficiently active” and “sufficiently active,” respectively.^28^

Exercise Barriers Scale: This instrument determined womens’ barriers to participate in exercise. Fourteen items strongly agree (4) to strongly disagree (0) were summarized ranging from 14 to 56. Higher scores indicated greater barriers to exercise.^29^

Patient Activation Measure (PAM‐13): This instrument measured the self‐management of health conditions by assessing knowledge, skills, and confidence.^30^, ^31^ The responses to 13 items were measured from strongly disagree (1) to agree strongly (4). The mean score of the PAM‐13 is the summation of total scores divided by the number of items responded, then this mean score was transformed into a 0–100 scale. The final scale was categorized into four activation levels according to scoring instructions: not believing activation is important (≤47.0); a lack of knowledge and confidence to take action (47.1–55.1); beginning to take action (55.2–67.0); and taking action (≥67.1).^31^

### 2.5 Statistical methods

Using descriptive statistical methods (mean, standard deviation, or range for continuous variables, and number and frequency for categorical variables), we summarized the socio‐demographic, anthropometric, clinical, and psychosocial characteristics of all participants, those who reported currently being discontent with their weight, and those who used one or more weight loss methods or programs during the past 12 months. The proportion of women who used each of the weight loss programs or methods ever or within the past 12 months was visualized in a stacked bar chart. Univariate analyses including the Pearson chi‐square tests for categorical variable and Student's *t*‐test for continuous variables were used. Multivariate logistic regression models adjusted for time since surgery and backward elimination methods were used to determine the factors associated with being discontent with body weight. All tests were two‐tailed, and a *p*‐value of 0.05 was considered significant. Data preparation and analyses were performed using SPSS version 27.0.

## 3 RESULTS

A total of 259/516 eligible women (50.2%) completed the long‐term follow‐up survey, at a median of 9 years (range 6–12 years) since surgery. At the time of survey, the average age of participants was 71 years (range 38–98 years), the mean self‐reported weight was 85 kilograms (kg) (range 69–98 kg), and 82% of the women were classified as overweight or obese (i.e., BMI≥25). Overall, 61 women (27%) reported they were content with their current weight, while 190 women (73%) were not content with current weight. Women who were discontent with their weight had lost significantly less weight (mean = −1.1) than those content (mean = −8.0; <0.001) since surgery (Table 1).

**TABLE 1:** Characteristics of long‐term endometrial cancer follow‐up survey participants and univariate analysis using chi‐square test for categorical or t‐test for continuous variables

	All participants, *n* = 259		Content with weight^a^, *n* = 61 (%)	Being discontent with weight^a^, *n* = 190 (%)	*p*‐value
Socio‐demographic characteristics					
Age in group (years), mean [SD]	71.39 [8.9]		75.55[8.95]	70.05 [8.52]	**0.000**
≤70	134 (51.7)		19 (14.3)	114 (85.7)	**0.000**
>71	125 (48.3)		42 (35.6)	76 (64.4)	
Education (n = 256)					0.198
Completed ≤12 years	168 (65.6)		44 (27.2)	118 (72.8)	
Completed >12 years	88 (34.4)		17 (19.8)	69 (80.2)	
Employment status as retired (n = 234)					0.452
Yes	158 (67.5)		32 (20.9)	121 (79.1)	
No	76 (32.5)		19 (25.3)	56 (74.7)	
Marital Status					0.174
Living with partner	175 (67.6)		37 (21.8)	133 (78.2)	
Living without partner	84 (32.4)		24 (29.6)	57 (70.4)	
Private Health Insurance					0.429
Yes	79 (30.5)		16 (21.1)	60 (78.9)	
No	180 (69.5)		45 (25.7)	130 (74.3)	
Birth Country					0.685
Australia	187 (72.2)		43 (23.6)	139 (76.4)	
Non‐Australia	72 (27.8)		18 (26.1)	51 (73.9)	
Anthropometric and weight loss program characteristics					
Weight at surgery, kg (mean [SD])	87.2 [21.5]		78.9 [24.1]	90. 4 [20.0]	**0.000**
Weight at follow‐up, kg (mean [SD])	84.5 [20.6]		69.2 [16.1]	89.1 [19.4]	**0.000**
Change of weight from surgery to follow‐up, kg (mean [SD])	−2.7 [11.7]		−8.0 [10.1]	−1.1 [10.1]	**0.000**
BMI (kg/m^2^), mean [SD] (*n* = 240)^b^	32.2 [8.54]		26.6 [7.3]	34.3 [7.4]	**0.000**
Normal weight (BMI <25)	43 (18.1)		26 (60.5)	17 (39.5)	**0.000**
Overweight and obese (BMI ≥25)	194 (81.9)		29 (15.0)	164 (85.0)	
Used one or more weight loss programs during past 12 months					
Never	31 (12.9)		19 (65.5)	10 (34.5)	
Ever or past 12 months^c^	210 (87.1)		38 (18.3)	170 (81.7)	**0.000**
Clinical characteristics (*n* = 237)					
Cardiovascular Disease					0.473
Yes	137 (52.8)		34 (25.0)	102 (75.0)	
No	100 (42.2)		21 (21.0)	79 (79.0)	
Diabetes Mellitus					0.943
Yes	48 (20.3)		11 (22.9)	37 (77.1)	
No	189 (79.7)		44 (23.4)	144 (76.6)	
Arthritis					0.490
Yes	36 (15.2)		10 (27.8)	26 (72.2)	
No	201 (84.8)		45 (22.5)	155 (77.5)	
Blood disorder					0.089
Yes	65 (27.4)		10 (15.6)	54 (84.4)	
No	172 (72.6)		45 (26.2)	127 (73.8)	
Other physical problem^d^					0.852
Yes	23 (9.7)		5 (21.7)	18 (78.3)	
No	214 (90.3)		50 (23.5)	163 (76.5)	
Hysterectomy type					0.750
Total Abdominal Hysterectomy	115 (44.4)		30 (27.0)	81 (73.0)	
Total Laparoscopic Hysterectomy	144 (55.6)		31 (22.1)	109 (77.9)	
Time since surgery (years), mean [SD]	8.55[1.29]		8.64 [1.40]	8.54 [1.25]	0.629
<9	124 (47.9)		30 (25.2)	89 (74.8)	0.750
≥9	135 (52.1)		31 (23.5)	101 (76.5)	
Other measurement					
Performance status (*n* = 252)					0.451
Fully active	119 (47.2)		26 (22.2)	91 (77.8)	
Not active	133 (52.8)		34 (26.4)	95 (73.6)	
Hospital Anxiety and Depression Scale‐ Anxiety, mean [SD]		4.4 [3.8]	4.1 [3.7]	4.5 [3.8]	0.496
Normal (0–7)	209 (81.6)		49 (24.3)	153 (75.7)	0.717
Anxiety (8–21)	47 (18.4)		10 (21.7)	36 (78.3)	
Hospital Anxiety and Depression Scale‐ Depression, mean [SD]	3.28 [3.0]		3.11 [3.61]	3.34 [2.82]	0.627
Normal (0–7)	225 (86.9)		51 (23.5)	166 (76.5)	0.778
Depression (8–21)	31 (12.1)		8 (25.8)	23 (74.2)	
Patient activation					0.134
Disengaged and overwhelmed/ Becoming aware, but still struggling/ taking action (<72.4)	182 (72.2)		46 (23.1)	130 (73.9)	
Maintaining behaviors and pushing further (≥72.4)	70 (27.0)		12 (17.1)	58 (82.9)	
Physical activity status					0.091
Sedentary/not active/ Insufficiently active (0–149)	87 (33.6)		13 (15.7)	70 (84.3)	
Sufficiently active (≥150)	158 (61.0)		39 (25.2)	116 (74.8)	
Functional Assessment of Cancer Therapy– General population, FACT‐GP (0–84)					
Physical well‐being (0–24)	21.1 [3.4]		21.1 [3.8]	21.0 [3.2]	0.947
Social well‐being (0–20)	14.7 [4.9]		15.5 [5.1]	14.5 [4.9]	0.161
Emotional well‐being (0–16)	14.1 [2.7]		14.5 [2.1]	14.0 [3.0]	0.165
Functional well‐being (0–24)	17.3 [5.5]		17.6 [5.8]	17.3 [5.4]	0.750
FACT–GP (0–84)	67.3 [12.4]		68.9 [13.3]	66.9 [12.2]	0.339
Exercise Barrier Scale (14–56)	36.5 [8.9]		36.7 [11.0]	36.7 [7.8]	0.984

Cronbach's Alpha (HADS: 0.957, anxiety: 0.905, depression: 0.922; Physical activity status: 0.911; Exercise barriers scale: 0.844; FACT‐GP, physical well‐being: 0.796, social well‐being: 0.784, emotional well‐being: 0.821, functional well‐being: 0.902).

Abbreviations: BMI, Body Mass Index; SD, Standard Deviation.

^a^ Number do not always round to 259 due to some missing responses.

^b^ The relative risks were 0.46 (95% CI: 0.32–0.67) for normal BMI and 4.02 (95% CI: 2.66–6.08) for overweight or obese BMI.

^c^ Ever (24.7%, *n* = 64), Past 12 months (56.4%, *n* = 146).

^d^ Included back pain, knee/hip surgery, spondylosis, urinary inconsistency, etc.

Bold values indicated statistically significant *p*‐value (<.05)

In univariate analysis, participants’ age, use of one or more weight loss methods, and being overweight or obese were found to be associated with discontent with weight, while other patient characteristics, such as co‐morbidities, time since surgery or whether surgery was using open or laparoscopic methods, performance status, anxiety, depression, patient activation, physical activity, and quality of life were not associated (Table 1). Adjusted for time since surgery, in multivariate analysis women who were discontent with their weight at long‐term follow‐up were more likely to be younger than 70 years (0.31, 95% CI: 0.14–0.67; *p* < 0.003) and using one or more weight loss methods ever or during the past 12 months (7.88, 95% CI: 2.73–22.75; *p* < 0.000) (Table 2). Four out of five women reported having ever used one or more weight loss programs or methods, with 146 women (56.3%) reporting they had used one or more weight loss programs or methods in the past 12 months (Table 1, Supplementary table).

**TABLE 2:** Association between being discontent with weight and characteristics adjusted logistic regression analyses

	Odds ratio (95% Confidence Interval)	*p*‐value
Age in group (years),		
≤70	1	
>71	0.31 (0.14–0.67)	**0.003**
Used one or more weight loss programs during past 12 months		
Never	1	
Ever or past 12 months	7.88 (2.73–22.75)	**0.000**
Time since surgery (years)		
<9	1	
≥9	1.98 (0.90–4.34)	0.087
Physical activity status		
Sedentary/not active/ Insufficiently active (0–149)	1	
Sufficiently active (≥150)	0.48 (0.20–1.13)	0.095

Bold values indicated statistically significant *p*‐value (<.05)

**FIGURE 1:**
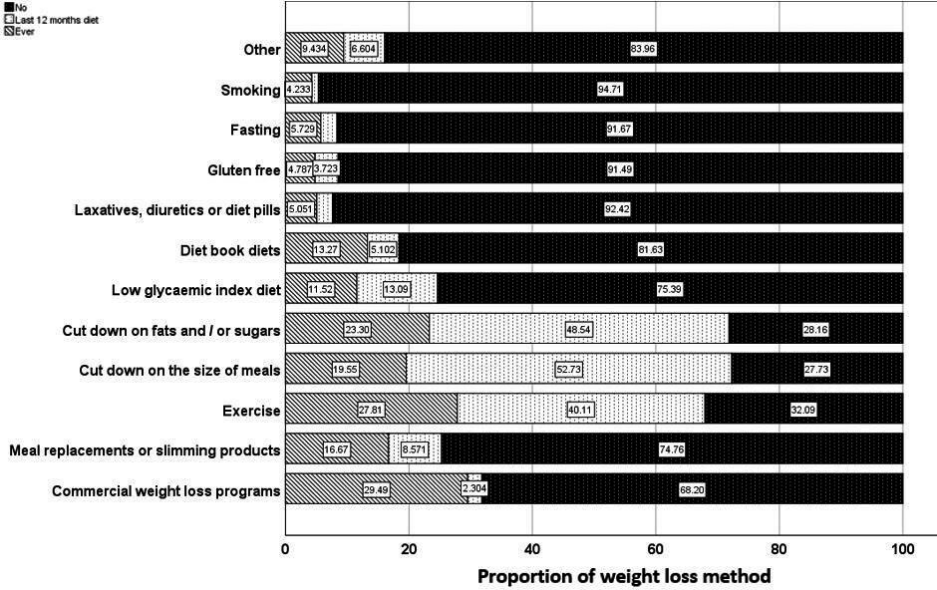
Proportion of women reporting specific weight loss methods according to whether they have never, recently (in the last 12 months) or ever used any weight loss methods

In the 12 months prior to completing the survey, a large proportion of women reported using exercise (40.1%), reducing meal intake (52.7%), and reducing fat/sugar intake (48.5%) for weight loss, while other methods were less commonly used. For example, only few women used fasting (1.8%) or designated weight loss programs (2.3%) during the past 12 months (Figure 1).

## 4 DISCUSSION

This analysis highlights the prevalence of long‐term EC survivors’ overweight (20.1%), obesity (54.8%), discontent with their weight (73%), and use of weight loss methods and programs (56%, used one or more in the past 12 months). Women who were discontent with the weight also reported lower weight loss since baseline. It further shows the role of sociodemographic, clinical, and psychosocial factors on women's perception of their current weight. In univariate and multivariate analyses, younger age, and having used one or more dietary methods over the past 12 months or ever were identified as factors associated with not being content with current weight (Table 2). Our study partially supports findings by Rowlands et al (2015), who reported that younger age at EC diagnosis was a significant predictor of having an unmet need (not necessarily related to weight) 3–5 years after treatment for EC.^32^

Overall, only 12% of the women reported never using a weight loss programs or methods in the past, with 56% using one or more methods over the past 12 months. We observed a strong association between recent use of weight loss methods and being discontent with weight. Exercise (48%), reduced meal (61%), or fat/sugar intake (59%) in the previous 12 months were the most commonly used methods reported by participants, while few women used fasting or designated weight loss programs. This may indicate that the weight loss efforts undertaken by many women were not sufficiently beneficial for them to feel content about their current weight. Similar to these results, Yoong et al^33^ found that exercise and diet changes, such as adherence to low‐fat and low‐calorie diets, were the most commonly used weight loss strategies among Australian patients in primary care. Prescription medications, detox diets, and fad/celebrity diets were among the least used methods.^33^

The optimal method to achieve weight loss is strongly debated, with some studies reporting that diets with low carbohydrate and high protein/fat content and/or diets that use intermittent fasting may be more effective for weight loss than other dietary efforts.^34^, ^35^ However, other studies reported no difference depending on which weight loss method was used even if it was aligned to a person's genotype, and emphasized rather the importance of a diet with high nutritional value.^36^ A current systematic review concluded that intentional weight loss is associated with reduced risk of EC; however, subsequent weight regain (weight cycling) increases its risk.^37^ This emphasizes that support for sustainable weight loss should be considered for patients diagnosed with EC,^38^ representing a good opportunity for targeted and personalized advice.

The most effective, but also most invasive form of weight management is through bariatric surgery. This method has been shown to lead to significant weight loss and consequent positive changes in endometrial pathologies, including resolution of atypical hyperplasia, lower risk of EC^39^ and decreased all‐cause mortality.^40^ Whether or not bariatric surgery should be discussed with, and would be acceptable for, women after EC diagnoses or treatment needs further study.

Given the findings of the present study, future supportive care programs seem urgently needed that assist women either to be content with their weight, or lose more weight, selecting the most adequate method or a nutritious diet most suitable to their lifestyle or household needs.^41^ Women diagnosed with gynecological cancers, particularly those classified as overweight or obese, have been shown to underestimate their weight status in self‐reports, but often are aware of the risks associated with weight gain, and report they would be receptive to weight management counselling from their healthcare providers.^42^

This study has considerable strength, being one of only a few to follow women long‐term after treatment of EC, and assessing a variety of potential factors associated with current weight and weight perceptions. These data provide evidence that issues related to weight are of considerable importance to women's perception of their health outcomes. They also provide evidence that women concerned about their weight and certain demographic or socioeconomic subgroups are more likely to use weight loss programs, and it needs to be studied if these differences persist if women were offered supportive care programs post‐treatment. Post‐treatment weight changes have been shown to impact survival in some cancer subgroups; for example, weight gain following treatment for breast cancer has been shown to negatively impact recurrence and survival,^43^ while weight changes after treatment for colon cancer did not significantly alter prognosis.^44^ Monitoring weight changes as well as women's perceptions of their weight following treatment for EC should therefore become an important part of post‐treatment surveillance.

A limitation of this study may be related to response bias, which is common for surveys sent by mail, with participation related to many factors including length of questionnaire, ethnicity, and cultural sensitivity to survey content.^45^ However, after accounting for women who had withdrawn or died since first enrolling into the LACE trial, the response rate in this study was over 50%, which is excellent given the women's age and surgery for some being more than 10 years ago. It is likely that those who did not participate may have had poorer quality of life or other health issues that affected their functionality, and therefore the findings likely represent those women with better well‐being.

In summary, our study provides evidence of weight perceptions, the types of weight control methods, and time frames in which women utilize them in long‐term survivors of EC. In light of the risks associated with weight gain, both physical and psychosocial, efforts to improve and counsel patient on their health status, particularly their weight, should become an integrated part of patient care after treatment for EC.

## CONFLICT OF INTEREST

The authors declare that there is no conflict of interest.

## AUTHOR CONTRIBUTION

Monika Janda, Peta Forder, Val Gebski, Andreas Obermair: Conceptualization, methodology, writing—review and editing; Saira Sanjida, Nigel Armfield: Data analysis. All authors: Approved final draft of manuscript.

## ETHICAL STATEMENT

The LACE trial (clinicaltrials.gov NCT00096408 and Australian New Zealand Clinical Trials Registry CTRN12606000261516) was approved by the ethics and governance committees of each study hospital and The University of Queensland (Approval 2006000368).

## Supporting information

Supplementary Material

## Data Availability

The data that support the findings of this study are available on request from the corresponding author. The data are not publicly available due to privacy or ethical restrictions.
