# Supplementary material for: Weight and weight control behaviors during long‐term endometrial cancer survivorship: Results of the Laparoscopic Approach to Cancer of the Endometrium long‐term follow‐up study
Source: Cancer Med. 2021 Jun 18;10(14):4896–904. doi: 10.1002/cam4.4032 (PMC8290251; doi:10.1002/cam4.4032)
Supplement: Supplementary file 1 — Supplementary Material [file CAM4-10-4896-s001.docx]

**Supplementary table**: Characteristics of long-term endometrial cancer follow-up survey participants Used at least one or more weight loss programs during past 12 months

|  | Used at least one or more weight loss programs during past 12 months,  n = 146 (%) |
| --- | --- |
| Socio-demographic characteristics | |
| Age, years |  |
| Mean [Standard Deviation] | 69.86 [8.8] |
| Min; max | 38; 91 |
| Age in group, years |  |
| ≤70 | 89 (70.1) |
| >71 | 57 (50.0) |
| Education |  |
| Completed ≤12 years | 86 (55.1) |
| Completed >12 years | 59 (71.1) |
| Employment status at follow-up |  |
| Retired | 94 (63.9) |
| Employed | 43 (58.1) |
| Marital Status |  |
| Living with partner | 99 (61.5) |
| Living without partner | 47 (58.8) |
| Private Health Insurance |  |
| Yes | 47 (61.8) |
| No | 99 (60.0) |
| Birth Country |  |
| Australia | 109 (62.3) |
| Non-Australia | 37 (56.1) |
| Anthropometric characteristics | |
| Weight at surgery, kg (mean [Standard Deviation]) | 89.99 [21.1] |
| Weight at follow-up, kg (mean [Standard Deviation]) | 87.00 [20.4] |
| Body Mass Index (mean, Standard Deviation) | 33.58 [7.5] |
| Body Mass Index (kg/m^2^) categories |  |
| Underweight-normal weight (<25) | 15 (35.7) |
| Overweight (25 - 29.9) | 34 (69.4) |
| Obese (≥30) | 91 (67.4) |
| Weight perceptions |  |
| Content with current weight | 19 (33.3) |
| Not content with current weight | 126 (70.0) |
| Clinical characteristics | |
| Comorbidities |  |
| Cardiovascular Disease | 73 (57.0) |
| Diabetes Mellitus | 26 (57.8) |
| Arthritis | 23 (67.6) |
| Respiratory disease | 19 (59.4) |
| Blood disorder | 40 (67.8) |
| Other | 14 (60.9) |
| Time since surgery, years (mean [Standard Deviation]) | 8.45 [1.2] |
| Hysterectomy type |  |
| TAH | 56 (53.8) |
| TLH | 90 (65.7) |
| Other measurements | |
| Performance status |  |
| Fully active | 77 (67.5) |
| Restricted in physically strenuous activity | 61 (59.8) |
| Ambulatory and capable of all or limited self-care | 8 (38.1) |
| Hospital Anxiety and Depression Scale- Anxiety | |
| Normal (0–7) | 118 (60.2) |
| Borderline anxiety (8–10) | 17 (68.0) |
| Anxiety (11-21) | 10 (55.6) |
| Hospital Anxiety and Depression Scale- Depression | |
| Normal (0–7) | 131 (61.5) |
| Borderline depression (8–10) | 11 (57.9) |
| Depression (11-21) | 3 (42.9) |
| Physical activity status |  |
| Sedentary/not active (0) | 7 (31.8) |
| Insufficiently active (1–149) | 38 (70.4) |
| Sufficiently active (≥150) | 96 (62.7) |
| Exercise barriers scale (14-56) |  |
| Mean Score | 37.24 [8.1] |
| Functional Assessment of Cancer Therapy– General population (0-84) | |
| Physical wellbeing (0–24) | 21.16 [3.2] |
| Social wellbeing (0–20) | 14.70 [4.7] |
| Emotional wellbeing (0–16) | 13.99 [3.0] |
| Functional wellbeing (0–24) | 18.11 [4.8] |
| FACT–GP (0–84) | 68.07 [11.72] |
| Patient activation |  |
| Disengaged and overwhelmed/ Becoming aware, but still struggling (≤55.1) | 8 (47.1) |
| Taking action (55.2–72.4) | 88 (59.1) |
| Maintaining behaviors and pushing further (≥72.4) | 48 (68.6) |
